# Molecular Dynamics Simulations of the miR-155 Duplex: Impact of Ionic Strength on Structure and Na^+^ and Cl^−^ Ion Distribution

**DOI:** 10.3390/molecules29174246

**Published:** 2024-09-07

**Authors:** Anna Rita Bizzarri

**Affiliations:** Biophysics and Nanoscience Centre, DEB, Università della Tuscia, Largo dell’Università, 01100 Viterbo, Italy; bizzarri@unitus.it; Tel.: +39-0761-357031

**Keywords:** microRNA, miR-155, molecular dynamics simulations, ion condensation, ionic strength

## Abstract

MiR-155 is a multifunctional microRNA involved in many biological processes. Since miR-155 is overexpressed in several pathologies, its detection deserves high interest in clinical diagnostics. Biosensing approaches often exploit the hybridization of miR-155 with its complementary strand. Molecular Dynamics (MD) simulations were applied to investigate the complex formed by miR-155 and its complementary strand in aqueous solution with Na^+^ and Cl^−^ ions at ionic strengths in the 100–400 mM range, conditions commonly used in biosensing experiments. We found that the main structural properties of the duplex are preserved at all the investigated ionic strengths. The radial distribution functions of both Na^+^ and Cl^−^ ions around the duplex show deviation from those of bulk with peaks whose relative intensity depends on the ionic strength. The number of ions monitored as a function of the distance from the duplex reveals a behavior reminiscent of the counterion condensation near the duplex surface. The occurrence of such a phenomenon could affect the Debye length with possible effects on the sensitivity in biosensing experiments.

## 1. Introduction

MicroRNAs (miRNAs) are a large family of noncoding RNAs, typically consisting of single strands of about 19–25 nucleotides. MiRNAs are present in different organisms, where they generally target protein-coding mRNAs at the post-transcriptional level, regulating complex patterns of gene expression [1,2,3]. Dysregulation of miRNAs, often manifesting as overexpression, is associated with several diseases, making them crucial biomarkers in diagnostics and clinical practice [4,5]. Consequently, detecting miRNAs, especially at low concentrations, is a significant objective in medical diagnostics. Among various detection methods, Electrochemical Impedance Spectroscopy (EIS) and Field Effect Transistor (FET) biosensors warrant particular attention [6]. These methods offer an excellent balance of sensitivity, speed, low cost, and ease of use [7,8]. These biosensors generally exploit a capture molecule (probe), commonly provided by the miRNA complementary single strand, which interacts with the target miRNA itself giving rise to the formation of a double strand through hybridization [9]. Ions are required to overcome the repulsion between their negatively charged phosphate backbones, then favoring the pairing of complementary RNA strands [10]. On the other hand, a high ionic strength limits the detection capability of these electrochemical biosensors since it shortens the so-called Debye length, which defines the active region through which electrons from the target can be transferred to the electrode surface, giving rise to the detection signal [11]. In this context, understanding the ion distribution around the miRNA duplex at different ionic strengths is crucial for interpreting experimental data from biosensors. We aim to investigate the ion distribution around the duplex formed by an miRNA and its complementary strand (Apt-155) in solution at different ionic strengths. Here, we applied a computational approach combining modeling and all-atom Molecular Dynamics (MD) simulations, allowing us to follow the dynamics of the whole system at the atomic level. The duplex, whose structure was initially derived through suitable modeling procedures, was placed in a box filled with water and varying amounts of Na^+^ and Cl^−^ ions to achieve ionic strengths ranging from 100 mM to 400 mM. This range was chosen to match the conditions commonly used in biosensing experiments, ensuring the stability of the duplex in connection with ion distribution [12]. We specifically focused on miR-155, a multifunctional miRNA that regulates cell differentiation, developmental stages, and the mammalian immune system [13]. MiR-155 is overexpressed or mutated in various malignant tumor cells, such as hepatocellular carcinoma, breast cancer, and colon cancer [14]. As a suitable biomarker for many types of cancers and other diseases, its detection is of high interest in diagnostics and screening [15,16]. Recently, we investigated the binding kinetics between miR-155 and its complementary strand by Surface Plasmon Resonance (SPR) and Atomic Force Spectroscopy (AFS) under conditions reminiscent of those commonly used in biosensing experiments and close to physiological conditions [17]. We found that miR-155 and Apt-155 form a duplex with a rather high affinity at the ionic strength of 300 mM, which represents a good compromise among the requirements to assure a stable duplex formation and a satisfactory detection level. Based on this, an understanding of the effective ion distribution around the duplex in relation to the ionic strength could help in interpreting results from biosensing experiments and could also provide valuable insights for the development of more effective biosensors.

## 2. Results

### 2.1. Structure of the Duplex

**Figure 1:**
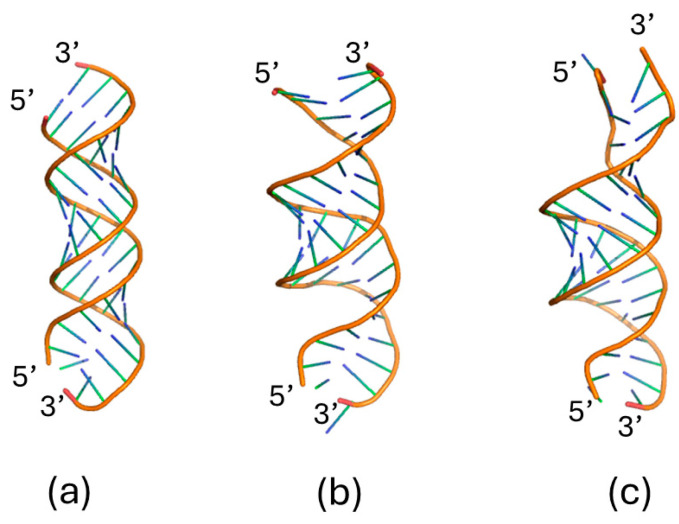
Graphical representation of (**a**) initial structure of the miR-155/Apt-155 duplex derived by the docking procedure, (**b**) the structure after equilibration used as initial configuration, and (**c**) final structure of the miR-155/Apt-155 duplex after a 500 ns run at a ionic strength of 100 mM.

Figure 1a shows the initial configurations of the miR-155/Apt-155 duplex, as derived by a docking procedure between the single-stranded modelled structure for miR-155 and Apt-155 (see Materials and Methods Section). Such a structure has been put at the center of a box filled with water molecules and a different number of ions (Na^+^ and Cl^−^) to reach an ionic strength within the 100–400 mM range; the system with only Na^+^ counterions for neutrality has been also investigated (named as the 0 mM ionic strength). The analyzed systems have been summarized in Table 1.

**Figure 2:**
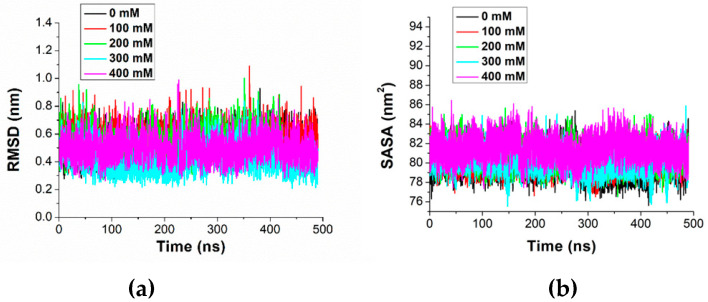
Representative temporal evolution of (**a**) RMSD and (**b**) SASA for the miR-155/Apt-155 duplex at different ionic strengths.

During equilibration, the modeled structure of the duplex undergoes a re-arrangement, as is emerging in Figure 1b. Successively, the dynamics of the miR-155/Apt-155 duplex systems, at different ionic strengths, have been followed for 500 ns in triplicate. By visual inspection, the duplex maintains its structure at all the ionic strength throughout the run; an example of the structure at the end of a run is shown in Figure 1c. Such a result is also witnessed by the temporal evolution of the RMSD, calculated using the structure of the duplex derived from the modelling as the reference. RMSDs exhibit fluctuations of about 0.08 nm around the initial value of 0.5 nm for all the ionic strengths (see Figure 2a). A similar behavior is also detected for SASA at the various ionic strengths (see Figure 2b). For both RMSD and SASA, no significant differences among the ionic strengths can be observed. This is confirmed by the averages and the corresponding standard deviations, evaluated over all the runs, reported in Table 1. Similarly, the RMSFs, the number of the intermolecular H-bonds, and the length of the duplex do not reveal any significant differences (see also Table 1). Globally, these results indicate that the duplex preserves its main structural properties during the dynamics in the analyzed range of ionic strengths.

### 2.2. Water and Ion Distribution around the Duplex

The environment around the duplex has been first investigated by analyzing the radial density function (RDF) of water and of the ions.

**Figure 3:**
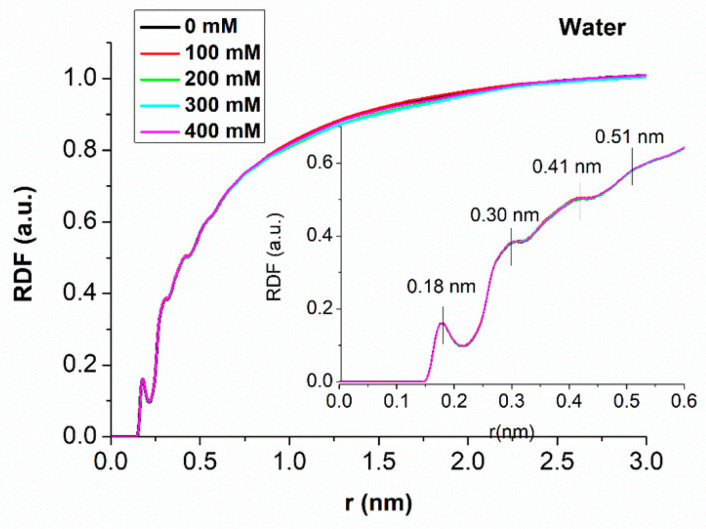
Normalized radial distribution function (RDF) of water at different ionic strengths as a function of the distance from the duplex. Inset: zoom of the region closer to the duplex. Each curve was obtained by averaging data over the three independent 500 ns long runs.

Figure 3 shows the normalized RDF curves of water as a function from the distance, r, of water molecules from the duplex nearest the atom at the different ionic strengths; a zoom of the region closer to the duplex being shown is in the inset. Each curve has been derived by averaging curves over the three independent runs. Notably, all the curves are found practically superimposed at all of the distances, with no differences among the three replicas. This means that the global behavior of water around the duplex does not change in the analyzed range of ionic strength. On the other hand, for distances below 2.5 nm from the duplex, the RDF values strongly deviate from one, which is the expected value for the bulk. More specifically, the RDFs are zero for distances up to 0.15 nm, then they undergo an increasing trend on which some peaks are superimposed. A first peak is found at 0.18 nm, followed by another at 0.22 nm. Smaller peaks are detected at 0.30 nm and 0.41 nm, with the corresponding minima at 0.33 nm and 0.44 nm, respectively. A very small bump is also detected at about 0.51 nm, after which the curves slowly increase to the bulk value, which is established and maintained for distances higher than 2.5 nm. These results are in a good agreement with those from other MD simulations of RNA in water, even if details of the RDF of water around macromolecules vary with the used Force Fields [18].

**Figure 4:**
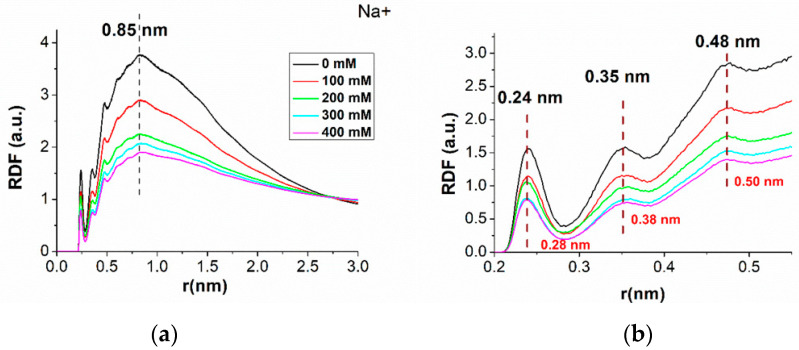
(**a**) Normalized radial distribution functions (RDFs) of Na^+^ ions, as a function of the distance from the duplex, at different ionic strengths; (**b**) zoom of the Figure in (a). Each curve was averaged over the three 500 ns long runs.

Figure 4a shows the normalized RDFs for the Na^+^ ion as a function of the distance r from the duplex at the different ionic strengths; a zoom of the region with r below 0.6 nm is shown in Figure 4b. Again, no variation among the replicas has been detected. All the curves are characterized by almost the same trend; however, lower values are detected as far as higher ionic strengths are considered. In more detail, the RDF curves are zero below 0.21 nm, indicating that no Na^+^ ions can be found in this region. We remark that water molecules have been found at a distance between 0.15 nm and 0.21 nm from the duplex. At distances higher than 0.21 nm, three main peaks appear in the RDF: the first is located at a distance 0.24 nm from the duplex, followed by a minimum at 0.28 nm; the second one is centered at 0.35 nm, with a minimum at 0.38; and the third is at 0.48 nm, with a minimum at 0.51 nm. It is interesting to note that the distance of the first minimum in the RDF of Na^+^ approximately corresponds to that at which the first maximum of the RDF is observed in water. Moreover, the RDF exhibits the absolute maximum, at 0.85 nm, after which a decreasing trend down to one can be detected.

**Figure 5:**
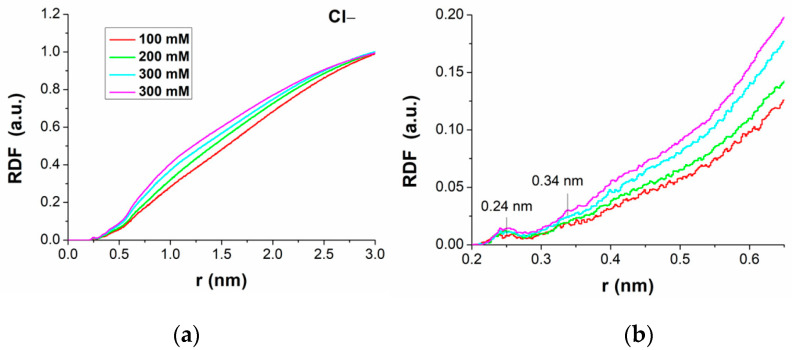
(**a**) Normalized radial distribution functions (RDFs) of Cl^−^ ions, as a function of the distance from the duplex, at different ionic strengths; (**b**) zoom of the Figure in a). Each curve was averaged over the three 500 ns long runs.

Figure 5a shows the normalized RDF of Cl^−^ ions as a function of the distance r of water molecules from the duplex at different ionic strengths, with a zoom of the region below 0.6 nm shown in Figure 5b. Very small values are registered in all cases; this is consistent with the fact that the negative charge of the duplex does not favor the presence of negative ions in its proximity. However, progressively higher values are detected with increasing ionic strengths. Below distances of 0.21 nm from the duplex, the RDF is zero, similar to that found for Na^+^ ions. At greater distances, the RDFs of Cl^−^ ions show a small peak located at 0.24 nm, followed by a minimum at 0.28 nm; this peak is found at the same position as the first one for Na^+^ ions; with a small bump appearing at 0.34 nm. At greater distances, there is an increasing trend that is more rapid for higher ionic strengths, indicating a progressive increase in the presence of Cl^−^ ions, as expected. The RDFs reach the value of one (bulk value) at distances of about 3.0 nm. According to a commonly used criterion [19], the first shell of ions around a charged polymer is defined from the first common minimum in both the Na^+^ and Cl^−^ RDF curves. In our case, it is located at about 0.28 nm, corresponding somewhat with the NMR data for such a shell falling within 0.4 nm from the DNA surface [20]. In this respect, it should however be mentioned that the distribution and the behavior of ions around DNA and around RNA could be slightly different [21]. Our results for the RDF of both Na^+^ and Cl^−^ ions around the duplex formed by miR-155 and its complementary strand are consistent with those found in the literature for MD simulations of duplexes formed by short oligonucleotides, which, however, are characterized by wide variability, depending on the Force Field used as well as the length of the molecule [21].

**Figure 6:**
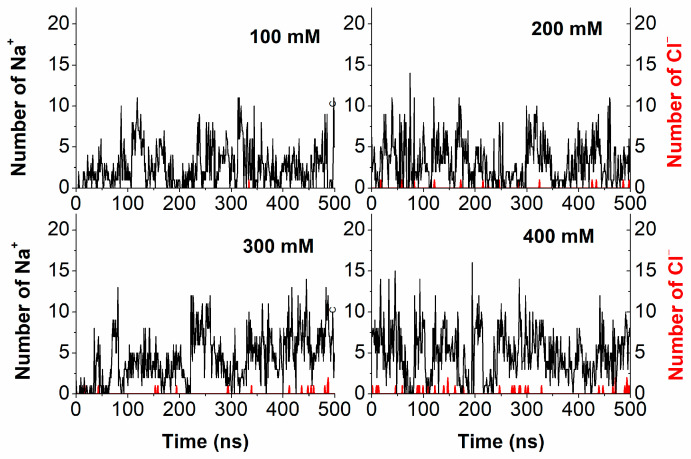
Representative temporal evolution of the number of Na^+^ (black lines) and Cl^−^ (red lines) ions within a region of 0.28 nm from the duplex at four ionic strengths.

Figure 6 shows representative trends in the number of Na^+^ (black line) and Cl^−^ (red lines) ions, as a function of time, found within 0.28 nm from the duplex for ionic strengths from 100 mM to 400 mM. In all of the cases, the number of Na^+^ ions exhibit rather wide fluctuations with values ranging from zero to progressively higher values as far as higher ionic strengths are concerned. At variance, Cl^−^ ions occasionally appear in the region, however, with a higher probability by increasing the ionic strength. These results are consistent with a relaxation time in the order of nanoseconds for ions in the first shell, as obtained from MD simulations for Na^+^ and Cl^−^ ions around oligonucleotides [18].

**Figure 7:**
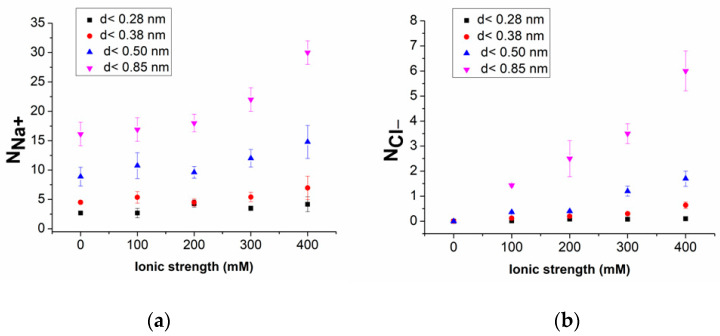
Average number of (**a**) Na^+^ ions (N_Na^+^_) and (**b**) Cl^−^ ions (N_Cl^−^_), as a function of the ionic strength at different distances from the duplex. Error bars indicate the variability over replicas.

We conducted detailed monitoring of the average number of ions at different distances from the duplex across various ionic strengths. Figure 7a,b show the number of Na^+^ (N_Na^+^_) and of Cl^−^ (N_Cl^−^_), respectively, as a function of the ionic strength near the duplex, with the variability among the replicas having been represented by the error bars. Concerning the Na^+^ ions, within distances of 0.28 nm and 0.38 nm from the duplex, N_Na^+^_ remains almost constant across the entire range of ionic strengths investigated. For wider distances, N_Na^+^_ stays constant below 200 mM but increases at higher ionic strengths, indicating a higher accumulation of sodium ions at elevated ionic strengths. For Cl^−^ ions, within 0.38 nm, N_Cl^−^_ is lower than one, consistent with the occasional passage of Cl^−^ ions in this region, as observed in Figure 6. At a distance of 0.50 nm, values around one are detected at higher ionic strengths. A marked increase in N_Cl^−^_ with the ionic strength is observed at the 0.85 nm region, though the values remain lower than those observed for Na^+^ ions. These results indicate that, close to the duplex surface, Na^+^ ions dominate over Cl^−^ ions, according to the negative charge of the duplex. Additionally, for ionic strengths below 200 mM, in the various regions around the duplex, the number of Na^+^ ions is almost constant, suggesting a sort of condensation. At the same time, Cl^−^ ions exhibit a more sporadic presence combined with a significant increase only at higher ionic strengths and larger distances from the duplex.

**Figure 8:**
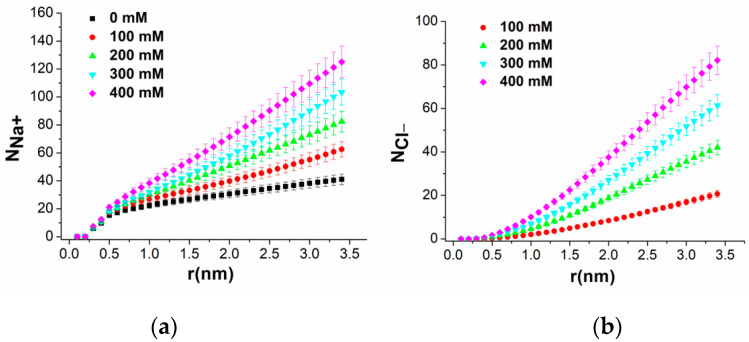
Average number of (**a**) Na^+^ ions (N_Na^+^_) and (**b**) Cl^−^ ions (N_Cl^−^_), as a function of distance from the duplex, at different ionic strengths. Error bars indicate the variability over replicas.

Figure 8a,b further analyze the behavior of Na^+^ and Cl^−^ ions as a function of the distance from the duplex, respectively, at different ionic strengths. Both N_Na^+^_ and N_Cl^−^_ increase with the distance from the duplex, but with a different behavior. Up to 0.5 nm, N_Na^+^_ follows a linear increasing trend that is almost the same for all ionic strengths. Beyond this distance, a progressive reduction from the initial linear trend is observed, more evident at lower ionic strengths. Conversely, a nonlinear increasing trend, with progressively higher slopes with the ionic strengths, is observed for N_Cl^−^_ consistently, with a larger number of Cl^−^ ions distancing themselves from the duplex surface. On the other hand, Cl^−^ ions display a more complex pattern, with their numbers significantly increasing at greater distances and higher ionic strengths.

**Figure 9:**
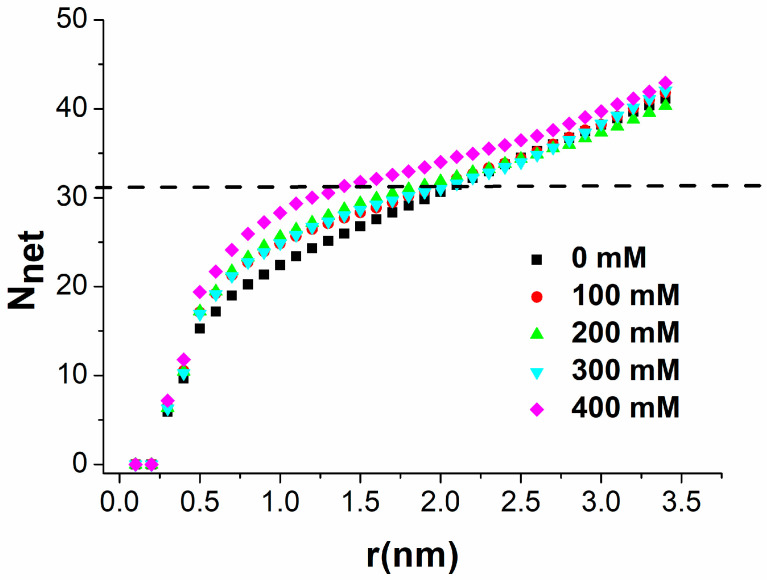
Total average number of positive charges, N_net_, (obtained from N_Na^+^_ – N_Cl^−^_) as a function of the distance from the duplex at different ionic strengths. The dashed horizontal black line indicates N_net_ equal to 31, corresponding to a fraction of 0.76 of the total charge.

Figure 9 shows N_net_ = N_Na^+^_ – N_Cl^−^_ as a function of the distance from the duplex, providing information about the number of net positive charges around the duplex, allowing the global visualization of the effective charges around the duplex.

For distances below 0.5 nm, N_net_ follows a linear trend which is practically the same in all the cases and is reminiscent of that observed for N_Na^+^_ (see Figure 8a). At greater distances, a deviation from linearity occurs, more markedly for low ionic strengths. Subsequently, N_net_ reaches a new linear regime that is quite independent of the ionic strength. Notably, this linear regime is established at a distance ranging from 2.0 to 2.5 nm, with progressively higher values as far as higher ionic strengths are concerned. These results suggest that three regions can be approximately identified around the duplex: (i) A region close to the duplex surface with practically only Na^+^ ions. The number of these ions follows a linear trend, whose slope is substantially independent on the ionic strength. (ii) A second region characterized by a transition toward a new linear regime, varying with the ionic strength. (iii) Finally, an external region is characterized by a linear trend, again almost independent of the ionic strength. The last behavior suggests a screening effect of the duplex charge and it can be discussed in connection with the Manning condensation theory for a charged polymer [22]. Briefly, the Manning theory predicts that if the so-called Manning parameter ξ exceeds a critical value, counterions (ions whose charge is opposite to that of the polymer) condensate in a confined region described by a condensation radius. For a charged cylinder polymer, the Manning parameter is given by ξ = λ_B_/b, where λ_B_ is the Bjerrum length and b the linear charge density of the polymer. In the presence of only monovalent counterions and at a high dilution, the critical value for the Manning parameter is one; with a corresponding fraction of ions of 1− 1/ξ, contained within the condensation radius, whose value depends on some parameters (such as concentration and pH) [22]. When a salt is present, a more complex behavior is registered; however, a condensation process with a related radius varying with the ionic strength has been predicted based on the Poisson–Boltzmann equation [23]. In our cases, the duplex can be satisfactorily modelled as a charged cylinder with a linear density of about 6e/nm (as also found for the double helix of oligonucleotides [24]). Since in water and at room temperature, the Bjerrum length λ_B_ is about 0.71 nm, a Manning parameter of ξ = 4.2 has been evaluated. Accordingly, in our system, ξ exceeds the critical value, and then a counterion condensation process is expected; the corresponding fraction of ions within the condensation radius is given by F_C_ = 1−1/ξ = 0.76. For a tentative estimation of the condensation radius, and by taking into consideration that F_C_ = 0.76, corresponding to a net charge of +31e, we have evaluated the distance at which such a charge is contained. The horizontal black dashed line plotted in Figure 9, related to N_net_ = 31, intercepts the N_net_ data at different distances ranging from 1.5 nm to 2.2 nm, varying with the ionic strength. We note that for ionic strengths below 200 mM, the charge fraction of 0.76 is contained within the same distance of about 2.2 nm. Such behavior is in good agreement with the presence of ion condensation, as predicted from the Manning theory.

Now, we wonder if the ion condensation around the duplex could have some implications in biosensing experiments. As a consequence of the condensation of Na^+^ ions around the duplex, the effective ionic strength around the duplex could be lower than expected. Consequently, a smaller number of ions could contribute to the effective ionic strength, leading to a longer Debye length. By taking into consideration the estimated condensation of 31 positive ions for the 300 mM ionic strength, the effective Debye length is 0.68 nm instead of 0.55 nm, with an increase of more than 20%. Such an effect could positively impact the biosensing capability since it allows charge detection at a greater distance from the electrode surface, and then a higher sensitivity even at a higher ionic strength. On the other hand, it could not be excluded that ion condensation around the duplex might simultaneously lead to a reduction in the polymer charge effectively detected. All these aspects provide some insights and warrant further experimental investigation.

## 3. Materials and Methods

### 3.1. Modelling 

The three-dimensional (3D) structure of the duplex was derived by the RNACOMPOSER software starting from the RNA oligonucleotide sequences for miR-155 (5′- uaa ugc uaa ucg uga uag ggg -3′) and the complementary strand Apt-155 (5′-ccc uau cac gau uag cau uaa-3′) [25]. All the figures for the biomolecules were created by Pymol and VMD [26,27].

### 3.2. Molecular Dynamics (MD) Simulations

MD simulations of the modelled miR-155/Apt-155 duplex in water were carried out by the GROMACS 2021.4 package [28], using the AMBER99SB-ILSD Force Field for RNA [29], and TIP3P for water [30]. The duplex molecule was centered in a rectangular box of edges 6.9 nm x 7.3 nm x 6.9 nm, filled with 10785 water molecules. A total of 41 water molecules were replaced with Na^+^ to assure neutrality, with these ions being mentioned as counterions, and the corresponding ionic strength being named as 0 mM. The box dimensions were fixed to have a distance between two adjacent duplexes similar to that expected in the experimental setups. Systems at different ionic strengths were analyzed, with the ionic strength defined as $1/2\sum_{i=1}^{n}c_{i}z_{i}^{2}$, where $c_{i}$ is the molar concentration of ions and$z_{i}$ is the charge number of ions. Systems at ionic strengths of 100 mM (72 Na^+^ and 31 Cl^−^ ions), 200 mM (103 Na^+^ and 62 Cl^−^ions), 300 mM (134 Na^+^ and 93 Cl^−^ions), and 400 mM (165 Na^+^ and 124 Cl^−^ ions), were obtained by replacing a defined number of water molecules with Na^+^ and Cl^−^ ions to reach the required conditions. H-bonds were constrained with the LINCS algorithm and a cut-off of 0.35 nm [31]. The Particle Mesh Ewald (PME) method [32,33] was applied to calculate the electrostatic interactions with a lattice constant of 0.12 nm. Simulation conditions were almost the same as those used by the authors in ref. [34,35]. The Periodic Boundary Conditions in the NPT ensemble were applied. The temperature was controlled by the Nosé–Hoover thermostat with a coupling time constant of 0.1 ps, while the Parrinello–Rahman extended ensemble, with a time constant of 1.0 ps, was used to control pressure [36,37]. Each system was minimized and then heated to 298 K with steps at 50 K, 100 K 150 K, and 250 K, and preliminarily submitted to 150 ns for relaxation. Successively, it was submitted to a 500 ns long MD trajectory, in triplicate for data analysis. Each replica was obtained by randomly changing the initial atom velocities using a Maxwell–Boltzmann distribution at the corresponding absolute temperature. The trajectories were monitored by analyzing the Root Mean Square Deviations (RMSDs), the Root Mean Square Fluctuation (RMSF), and the solvent accessible surface area (SASA), through the GROMACS package tools [38]. The length of the duplex was evaluated by measuring the distance between C3 G21 of miR-155 and C3 A42 of Apt-155. Furthermore, intermolecular H-bonds between miR-155 and its Apt-155 were also monitored in time by applying the geometrical criterion, assuming a donor–acceptor distance of 0.35 nm and the hydrogen donor–acceptor angle of 30°. The radial distribution function (RDF) of both water and ions around the duplex was calculated by the subroutines in GROMACS by taking into consideration the nearest N atom from the duplex.

## 4. Conclusions

MD simulations conducted on a negatively charged duplex formed by miR-155 and its complementary single strand, in aqueous solution with varying ionic strengths, employing Na^+^ and Cl^−^ ions, allowed us to investigate both the structure and the dynamics of the duplex and the behavior of these surrounding ions. The results indicated that the structure of the duplex, after equilibration, remained stable across different ionic strengths. Key parameters such as the RMSF, SASA, and number of intermolecular H-bonds showed negligible changes with varying ionic strengths. The RDFs of Na^+^ and Cl^−^ ions showed peaks at well-defined distances from the duplex, with the same features across different ionic strengths. The analysis revealed a notable condensation of Na^+^ ions around the duplex, consistent with the Manning condensation model observed in charged polymers. The occurrence of such a phenomenon suggests a reduction in the number of free ions contributing to the ionic strength, with a consequential decrease in the Debye length. This could lead to an enhancement of the biosensing capabilities for devices in which miRNAs are immobilized on electrodes. These findings highlight the importance of considering ionic strength in relation to the ion distribution, with this helping in the design and optimization of miRNA-based biosensors.

## Figures and Tables

**Table 1:** Selected properties of the duplex in water and with Na^+^ and Cl^−^ ions at different ionic strengths. The averages and standard deviations (in parentheses) have been obtained from three 500 ns long replicas.

Ionic strength	RMSD (nm)	SASA (nm^2^)	RMSF (nm)	H-bonds	Length (nm)
0 mM	0.52 (0.08)	80 (1)	0.24 (0.08)	60 (4)	6.6 (0.3)
100 mM	0.55 (0.09)	80 (1)	0.28 (0.10)	61 (4)	6.9 (0.4)
200 mM	0.54 (0.09)	81 (1)	0.26 (0.10)	61 (4)	6.9 (0.3)
300 mM	0.49 (0.09)	80 (1)	0.23 (0.06)	61 (4)	6.7 (0.3)
400 mM	0.51 (0.09)	82 (1)	0.26 (0.10)	61 (4)	6.5 (0.3)

## Data Availability

Data are available on request.

## References

[1] Bartel D.P. (2004). MicroRNAs: Genomics, Biogenesis, Mechanism, and Function. Cell.

[2] Ambros V. (2004). The Functions of Animal MicroRNAs. Nature.

[3] Hammond S.M. (2015). An Overview of MicroRNAs. Adv. Drug Deliv. Rev..

[4] Macfarlane L.A., Murphy P.R. (2010). MicroRNA: Biogenesis, Function and Role in Cancer. Curr. Genomics.

[5] Vaghf A., Khansarinejad B., Ghaznavi-Rad E., Mondanizadeh M. (2022). The Role of MicroRNAs in Diseases and Related Signaling Pathways. Mol. Biol. Rep..

[6] Ouyang T., Liu Z., Han Z., Ge Q. (2019). MicroRNA Detection Specificity: Recent Advances and Future Perspective. Anal. Chem..

[7] Lowe B.M., Sun K., Zeimpekis I., Skylaris C.-K., Green N.G. (2017). Field-effect Sensors—From pH Sensing to Biosensing: Sensitivity Enhancement Using Streptavidin-Biotin as a Model System. Analyst.

[8] Sung D., Koo J. (2021). A Review of BioFET’s Basic Principles and Materials for Biomedical Applications. Biomed. Eng. Lett..

[9] Hwang M.T., Heiranian M., Kim Y., You S., Leem J., Taqieddin A., Faramarzi V., Jing Y., Park I., van der Zande A.M. (2020). Ultrasensitive Detection of Nucleic Acids Using Deformed Graphene Channel Field Effect Biosensors. Nat. Commun..

[10] Gong P., Levicky R. (2008). DNA Surface Hybridization Regimes. Proc. Natl. Acad. Sci. USA.

[11] Tadmor R., Hernández-Zapata E., Chen N., Pincus P., Israelachvili J.N. (2002). Debye Length and Double-layer Forces in Polyelectrolyte Solutions. Macromolecules.

[12] Poghossian A., Schoning M.J. (2014). Label-Free Sensing of Biomolecules with Field-Effect Devices for Clinical Applications. Electroanalysis.

[13] Faraoni I., Antonetti F.R., Cardone J., Bonmassar E. (2009). miR-155 Gene: A Typical Multifunctional MicroRNA. Biochim. Biophys. Acta.

[14] Due H., Svendsen P., Bødker J.S., Schmitz A., Bøgsted M., Johnsen H.E., El-Galaly T.C., Roug A.S., Dybkær K. miR-155 as a Biomarker in B-Cell Malignancies. Biomed. Res. Int..

[15] Mattiske S., Suetani R.J., Neilsen P.W., Callen D.F. (2012). The Oncogenic Role of miR-155 in Breast Cancer. Cancer Epidemiol. Biomarkers Prev..

[16] Hou Y., Wang J., Wang X., Shi S., Wang W., Chen Z. (2016). Appraising MicroRNA-155 as a Noninvasive Diagnostic Biomarker for Cancer Detection: A Meta-Analysis. Medicine (Baltimore).

[17] Botti V., Lavecchia di Tocco F., Cannistraro S., Bizzarri A.R. (2023). Hybridization Kinetics of miR-155 on Gold Surfaces as Investigated by Surface Plasmon Resonance and Atomic Force Spectroscopy. ACS Omega.

[18] Kührová P., Otyepka M., Šponer J., Banáš P. (2013). Are Waters around RNA More than Just a Solvent? – An Insight from Molecular Dynamics Simulations. J. Chem. Theory Comput..

[19] Feig M., Pettitt B.M. (1999). Sodium and Chlorine Ions as Part of the DNA Solvation Shell. Biophys. J..

[20] Strzelecka T.E., Rill R.L. (1992). 23Na NMR of Concentrated DNA Solutions: Salt Concentration and Temperature Effects. J. Phys. Chem..

[21] Cruz-León S., Schwierz N. (2022). RNA Captures More Cations than DNA: Insights from Molecular Dynamics Simulations. J. Phys. Chem. B.

[22] Manning G.S. (1978). The Molecular Theory of Polyelectrolyte Solutions with Applications to the Electrostatic Properties of Polynucleotides. Q. Rev. Biophys..

[23] Bizzarri A.R., Cametti G., Di Biasio A. (1988). Counterion Accumulation in Rod-Like Polyelectrolyte Solutions with Added Salt and Manning’s Condensation Theory. Ber. Bunsenges. Phys. Chem..

[24] Phillips R., Kondev J., Theriot J. (2009). Physical Biology of the Cell.

[25] Popenda M., Szachniuk M., Antczak M., Purzycka K.J., Lukasiak P., Bartol N., Blazewicz J., Adamiak R.W. (2012). Automated 3D Structure Composition for Large RNAs. Nucleic Acids Res..

[26] Guex N., Peitsch M.C. (1997). SWISS-MODEL and the Swiss-PdbViewer: An Environment for Comparative Protein Modeling. Electrophoresis.

[27] Humphrey W.F., Dalke A., Schulten K. (1996). VMD—Visual Molecular Dynamics. J. Mol. Graph..

[28] Lindahl A., Abraham M., Hess B., van der Spoel D. (2021). GROMACS 2021 Source Code (Version 2021).

[29] Lindorff-Larsen K., Piana S., Palmo K., Maragakis P., Klepeis J.L., Dror R.O., Shaw D.E. (2010). Improved Side-chain Torsion Potentials for the AMBER ff99SB Protein Force Field. Proteins.

[30] Jorgensen W.L., Chandrasekhar J., Madura J.D., Impey R.W., Klein M.L. (1983). Comparison of Simple Potential Functions for Simulating Liquid Water. J. Chem. Phys..

[31] Hess B., Bekker H., Berendsen H.J., Fraaije J.G. (1997). LINCS: A Linear Constraint Solver for Molecular Simulations. J. Comput. Chem..

[32] Kholmurodov K., Smith W., Yasuoka K., Darden T., Ebisuzaki T. (2000). A Smooth-Particle Mesh Ewald Method for DL_POLY Molecular Dynamics Simulation Package on the Fujitsu VPP700. J. Comput. Chem..

[33] Darden T., York D., Pedersen L. (1993). Particle Mesh Ewald: An N⋅log(N) Method for Ewald Sums in Large Systems. J. Chem. Phys..

[34] Botti V., Cannistraro S., Bizzarri A.R. (2022). Interaction of miR-155 with Human Serum Albumin: An Atomic Force Spectroscopy, Fluorescence, FRET, and Computational Modelling Evidence. Int. J. Mol. Sci..

[35] Bizzarri A.R. (2022). Conformational Heterogeneity and Frustration of the Tumor Suppressor p53 as Tuned by Punctual Mutations. Int. J. Mol. Sci..

[36] Nosé S. (1984). A Unified Formulation of the Constant Temperature Molecular Dynamics Methods. J. Chem. Phys..

[37] Parrinello M., Rahman A. (1981). Polymorphic Transitions in Single Crystals: A New Molecular Dynamics Method. J. Appl. Phys..

[38] Abraham M.J., Murtola T., Schulz R., Páll R., Smith  J.C., Hess B., Lindahl E. (2015). Gromacs: High Performance Molecular Simulations through Multi-level Parallelism from Laptops to Supercomputers. SoftwareX.

